# Cryoballoon ablation for paroxysmal atrial fibrillation in a case of persistent left superior vena cava

**DOI:** 10.1186/s12872-018-0789-1

**Published:** 2018-03-13

**Authors:** Shiwei Huang, Binglin Pan, He Zou, Wei Lin

**Affiliations:** Department of Cardiology, Wenzhou People’s Hospital, Wenzhou, Zhejiang Province 325000 China

**Keywords:** Atrial fibrillation, Persistent left superior vena cava, Cryoballoon ablation

## Abstract

**Background:**

Atrial fibrillation (AF) usually originates from pulmonary veins (PVs) but can also be caused by pulmonary veins outside, such as the coronary sinus (CS), the superior vena cava (SVC), and the ligament of Marshall.

**Case presentation:**

A 69-year-old male with a history of palpitations for 10 years was referred to our institute because of its recurrence for half a day. A dynamic electrocardiogram revealed sinus rhythm (SR) and paroxysmal AF. Echocardiography demonstrated normal cardiac structure, and physical examination results were unremarkable. However, computed tomography angiography (CTA) showed a persistent left superior vena cava (LSVC) but no indication of thrombosis in the left atria. A cryoablation catheter was inserted into the PV. After the PV was successfully isolated, AF was still observed. After cardioversion was synchronized, SR was detected, but AF occurred again in less than a minute. Finally, we observed ectopic atrial electrical activity originating from the LSVC and successfully ablated it.

**Conclusions:**

An LSVC may be a substrate for initiating or perpetuating atrial arrhythmia. Cryoballoon ablation can help treat AF originating from the LSVC.

## Background

Atrial fibrillation (AF) usually originates from pulmonary veins (PVs) but can also be caused by pulmonary veins outside, such as the coronary sinus (CS), the superior vena cava (SVC), and the ligament of Marshall. This article reports a case of successful cryoballoon ablation for AF in a persistent left superior vena cava (LSVC).

## Case presentation

**Fig. 1:**
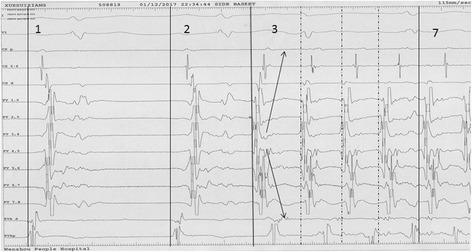
Recordings of the surface electrocardiogram (above) and intracardiac electrocardiogram (below). Intracardiac recordings of a quadripolar ablation catheter (bipolar distal: RVAd; proximal: RVAp) and recordings of a spiral catheter (PV 1,2 to PV 7,8). Both catheters are located in positions similar to those shown in Fig. 2. The first (1) and second (2) beats show activation during sinus rhythm (SR). The third (3) to last (7) beats correspond to AF, and in these beats, the activation of PV 3,4 and PV 4,5 likely occurs first

**Fig. 2:**
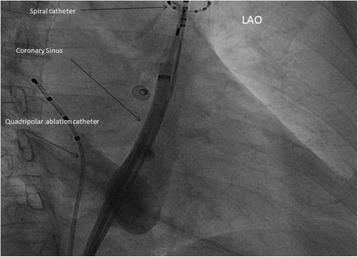
LAO: Coronary sinus and left superior vena cava (LSVC) course demonstrated through angiography. Mapping of the atrial trigger activity in LSVC by using a spiral decapolar catheter. A quadripolar ablation catheter was placed in the right vena cava

A 69-year-old male with a history of palpitations for 10 years was referred to our institute because of its recurrence for half a day. Dynamic electrocardiogram revealed SR and paroxysmal AF. Echocardiography demonstrated normal cardiac structure, and physical examination results were unremarkable. However, CTA showed a persistent LSVC but no indication of thrombosis in the left atria. The decapolar catheter was introduced into the CS via the left femoral vein. However, we observed that the line was abnormal. Radiography suggested that the left superior vena cava and CS led into the right atrium. An interatrial septum puncture was performed under the guidance of a standard measuring electrode (Achieve), and a cryoablation catheter was inserted into the PV. After the PV was successfully isolated, AF was still observed. After cardioversion was synchronized, SR was detected, but AF occurred again in less than a minute. We repeated the procedure thrice but obtained similar results. A quadripolar ablation catheter was inserted retrograde through the CS into the LSVC. Premature atrial complexes and short paroxysmal atrial tachycardia were observed before the onset of AF, and the vena cava potential was observed before atrial potential. Other aspects of the atrial electrical activity and body surface electrocardiogram (ECG) included ectopic P waves, and the CS excitation order was from distal to proximal (Figs. 1 and 2). This finding suggested ectopic excitation originated in the LSVC. Using the CS pacemaker, we conducted radiofrequency catheter ablation in parts of the LSVC with an enlarged coronary vein distal border, and our ablation target was the vena cava potential with a relatively early and strong amplitude. SR was recorded during discharge, the vena cava potential was delayed and thus disappeared completely.

## Discussion

Thoracic veins, including PVs and SVC, coronary veins, and the ligament of Marshall have been regarded as a substrates for initiating or perpetuating atrial arrhythmias. The presence of LSVC occurs in approximately 0.3%- 0.5% of the general population [1]. Since the LSVC is also a thoracic vein, it seems reasonable that the LSVC can play an important role in the pathogenesis of atrial fibrillation [2, 3]. As in this case, we observed that the AF originated in the LSVC. To ablate AF that originated from the LSVC, Hsu et al. [4] isolated the connections of the LA-LSVC and CS-LSVC. In this case, we would ring the measuring electrodes placed on the LSVC and examine the potential with a relatively early and strong amplitude. According to radiography, the location was at the opening of the SVC, considering that this location was higher than and farther from the coronary artery, we did not perform coronary angiography. The LSVC should be examined not only as an anomalous structure but also as a substrate for AF. The patient and his family agreed and signed a surgical consent form before electrophysiology and cryoballoon ablation were performed.

## Conclusions

LSVC may be a substrate for initiating or perpetuating atrial arrhythmia. Cryoballoon ablation can help treat AF originating from the LSVC.

## Abbreviations

AF: Atrial fibrillation
CS: Coronary sinus
CTA: Computed tomograghy angiography
LSVC: Left superior vena cava
PVs: Pulmonary veins
SVC: Superior vena cava
